# Recurrent Testicular Torsion After Prior Surgical Fixation: A Two-Case Series and Technical Insight into Orchidopexy Failure

**DOI:** 10.3390/jcm15135231

**Published:** 2026-07-04

**Authors:** Julia Katharina Peters, Philipp Nikolaus Haid, Maximilian Pallauf, Lukas Lusuardi, Peter Törzsök

**Affiliations:** 1 Department for Urology and Andrology, University Hospital Salzburg, 5020 Salzburg, Austria; p.haid@salk.at (P.N.H.); m.pallauf@salk.at (M.P.); l.lusuardi@salk.at (L.L.); 2 Faculty of Health and Sport Sciences, Széchenyi István University, 9026 Győr, Hungary; torzsok.peter@gmail.com

**Keywords:** case series, recurrent testicular torsion, surgical fixation

## Abstract

**Background**: Testicular torsion is a urological emergency that requires prompt diagnosis and surgical intervention to preserve testicular viability. Bilateral orchidopexy is widely regarded as the definitive treatment for the prevention of recurrence. **Cases**: We report two rare cases of recurrent testicular torsion despite prior surgical fixation. Both patients had previously undergone orchidopexy and presented with acute scrotal pain. Surgical exploration confirmed torsion in both cases, despite macroscopically intact fixation sutures. Intraoperative findings suggested that recurrence resulted from insufficient restriction of mobility rather than suture failure. Revision orchidopexy with modified multi-point fixation was performed, resulting in successful detorsion and the preservation of testicular perfusion. **Results**: These cases highlight that orchidopexy does not universally prevent recurrent torsion and emphasize the critical importance of surgical technique, including suture placement, orientation, and number of fixation points. Increased awareness of potential re-torsion is essential, even in previously pexied patients. Improved standardization of fixation techniques may help reduce the risk of recurrence.

## 1. Introduction

Testicular torsion is a time-sensitive surgical emergency that most commonly affects adolescent males, with a peak incidence between 12 and 25 years of age.

The primary anatomical risk factor for intravaginal testicular torsion is the bell-clapper deformity, an abnormal attachment of the tunica vaginalis that allows the testis, epididymis, and distal spermatic cord to rotate freely within the hemiscrotum. This increased rotational mobility predisposes the testis to torsion. Because the anomaly is frequently bilateral, bilateral orchidopexy following surgical detorsion remains the standard treatment to prevent recurrence.

As shown in refs. [1,2], orchidopexy is considered definitive, with recurrence after fixation being exceptionally rare [3,4,5]. The recent literature has highlighted the importance of surgical techniques in orchidopexy, with a particular focus on suture material, number of fixation points, and orientation. However high-level evidence and standardized recommendations for optimal fixation technique are still lacking, particularly with respect to suture configuration and prevention of rotational mobility [5,6,7]. We report two cases of recurrent torsion in previously pexied testicles, raising concern regarding technique-dependent failure.

Both patients provided written consent for the reporting of their cases and the use of intraoperative photographs.

## 2. Case Presentation

**Figure 1:**
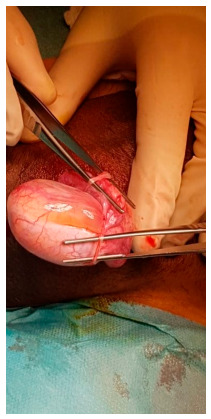
Case 1: Intact sutures from the first orchidopexy.

**Figure 2:**
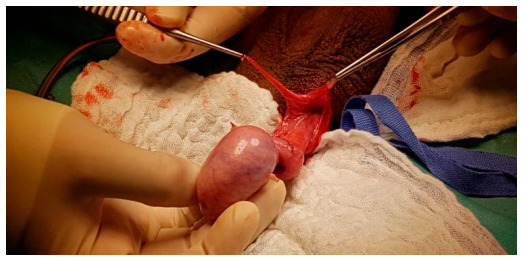
Case 1: Transected old pexy sutures with visible scar tissue.

Case 1: A 16-year-old male presented to our hospital in May 2020 with acute left scrotal pain. He had previously experienced right-sided testicular torsion in September 2019, which had been surgically managed at an outside institution with bilateral orchidopexy. Review of the operative report confirmed that a bilateral two-point orchidopexy had been performed using polydioxanone (PDS) sutures. On presentation in May 2020, approximately eight months after the primary procedure, surgical exploration confirmed recurrent left-sided torsion despite the presence of two macroscopically intact lateral fixation sutures from the previous orchidopexy. The findings were documented photographically, and the images demonstrate the persistence of the original fixation sutures (Figure 1 and Figure 2). Despite remaining visible and intact, the sutures had failed to adequately restrict rotational mobility of the testis. Revision orchidopexy was therefore performed using reinforced three-point fixation with non-absorbable sutures, resulting in successful detorsion and preservation of testicular perfusion.

**Figure 3:**
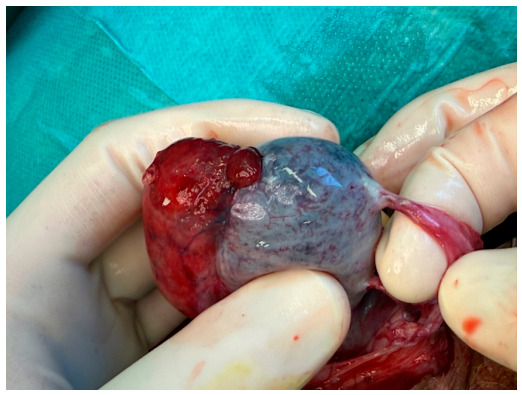
Case 2: Intraoperative image showing intact longitudinal prior pexy sutures in situ.

**Figure 4:**
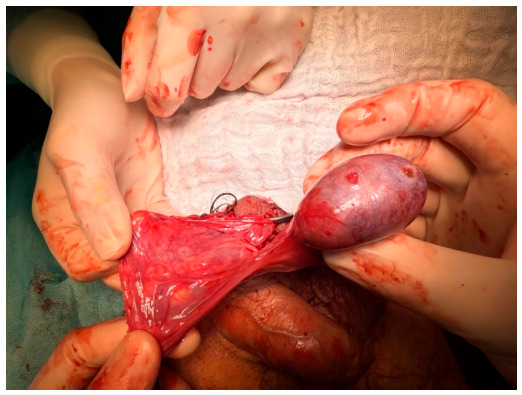
Case 2: Transected longitudinal sutures with scar tissue and reperfused testis following detorsion.

Case 2: A 19-year-old male experienced right-sided torsion in March 2000 and underwent detorsion with bilateral orchidopexy in our hospital. No complications were reported, and follow-up was uneventful. In May 2025, now aged 44, he again presented with left scrotal pain, which had been persisting for three days. An ultrasound of the left testis indicated the absence of blood perfusion, and acute surgical exploration of the testis revealed left-sided torsion with the testis rotated 360 degrees, despite a prior orchidopexy. Interestingly, the left testicle was pexied at two longitudinal points, likely to allow axial rotation. The old sutures were still visible, indicating that suture failure was not the cause but rather inadequate anchoring. Intraoperative findings also revealed scarred sites of previous fixation (Figure 3 and Figure 4). Detorsion was successful, and a new fixation with three-point sutures was performed with non-resolvable sutures. Furthermore, the contralateral testis (the one originally twisted in 2000) was also re-pexied to prevent any future recurrence. Although the patient had presented with a 72 h delay, the visual recovery of perfusion was sufficient to justify organ preservation. Consequently, a micro-biopsy was not performed to avoid further trauma to the recovering tissue. Post-operative follow-up at two weeks included a Doppler ultrasound, which confirmed excellent bilateral testicular perfusion. Subsequent routine screening 17 months later demonstrated continued stable perfusion on both sides.

## 3. Discussion

Recurrent torsion despite prior orchidopexy is exceptionally rare but has been documented in the literature. The choice of suture material appears to be a significant factor in orchidopexy failure. In the systematic review by van Welie et al., 84% of patients had initially been pexied with absorbable sutures [6]. Historically, it was believed that absorbable sutures would induce an inflammatory response leading to permanent adhesions; however, research indicates that these often result in only fine adhesions that fail to restrict rotational movement once the suture has dissolved [8]. While newer publications recommend three-point fixation with non-absorbable sutures, such as the BURST consensus, recurrence can occur even with permanent material if the technical configuration is insufficient, as seen in our two cases, where the sutures remained macroscopically intact but allowed axial rotation [6,9].

Case 2 is particularly instructive. It demonstrates that longitudinal fixation fails to prevent rotation around the vertical axis of the spermatic cord, which is the primary pathophysiology of torsion.

The clinical course of Case 2 is exceptional due to the patient’s age and the 72 h duration of symptoms. Testicular torsion primarily affects adolescents, with a peak incidence between 12 and 25 years. A presentation at 44 years, more than two decades after the initial orchidopexy, is remarkably rare. A presentation at this age, 25 years after the initial fixation, is exceptionally rare and significantly exceeds the age range reported in the systematic review by van Welie et al. (5–35 years) [6].

The exceptionally long interval of approximately 25 years between primary orchidopexy and recurrent torsion raises the possibility that long-term tissue remodeling, scar maturation, or other anatomical changes may have contributed to recurrent testicular mobility. However, the mechanisms underlying such delayed recurrences remain poorly understood. To date, the literature offers limited insight into whether age-related anatomical changes or long-term remodeling processes influence the risk of recurrent torsion after orchidopexy. This case underscores the necessity of maintaining a high index of suspicion for torsion, regardless of the patient’s age or surgical history.

Furthermore, the viability of the testis in Case 2 challenges established clinical thresholds. The standard literature defines a critical 4–6 h window for optimal salvage, with success rates falling below 10% after 24 h [6,10]. Notably, the systematic review by van Welie et al. reported zero successful salvage cases when pain persisted for more than 24 h [6].

This extraordinary viability may be explained by the specific nature of the torsion. Intraoperatively, the testis was found to be rotated 360 degrees. In contrast to more severe twists (e.g., 720° or 1080°), a 360° rotation may result in incomplete or intermittent vascular compromise rather than total occlusion. This suggests that even with persistent pain, the torsion may involve periods of partial detorsion or residual arterial flow, preserving tissue over a longer interval.

Our finding of immediate macroscopic reperfusion without the need for biopsy demonstrates that viability is possible even in late-presenting cases. This clinical decision was further validated by the long-term success of the procedure. Stable perfusion was confirmed via Doppler ultrasound at the 17-month follow-up, justifying the decision to prioritize salvage over orchiectomy.

Current guidelines offer little standardization regarding suture placement, number, or technique. Neither the European Association of Urology (EAU) nor the American Urological Association (AUA) guidelines provide explicit recommendations on the choice of suture material or specific fixation technique. This gap in guidance likely contributes to the technical failures described in the literature [11,12].

However, a consensus document from the British Association of Urological Surgeons (BAUS) and the British Urology Researchers in Surgical Training (BURST) favors the use of non-absorbable sutures and multi-point fixation, preferably three- or four-point fixation. The consensus specifically recommends placing sutures on the medial, lateral and inferior surfaces of the testis to ensure stability. Our cases underscore the clinical necessity of the technical standards. Specifically, our findings suggest that fixation must not be limited to the longitudinal axis. To effectively restrict axial rotation around the vertical axis of the spermatic cord, surgeons should include sutures in the transverse axis. This multi-axial approach enhances stability and directly addresses the mechanical failure observed in our series, where macroscopically intact longitudinal sutures still allowed for a 360-degree torsion to minimize the risk of recurrence, although robust comparative data remain limited [10]. These cases underscore the need for enhanced surgical awareness and possibly revised technical standards in orchidopexy procedures. We suggest that fixation should not be limited to the longitudinal axis but should also include sutures placed in the transverse axis, thereby restricting axial rotation more effectively and enhancing stability.

## 4. Conclusions

Recurrent testicular torsion (RTT) after orchidopexy is rare but can occur despite macroscopically intact sutures. Our cases highlight that longitudinal fixation alone is insufficient to prevent rotation around the vertical axis of the spermatic cord. Crucially, Case 2 demonstrates that testicular salvage is possible even after a 72 h ischemic interval, directly challenging the 24 h limit suggested by current systematic reviews. To minimize recurrence, we recommend a standardized three-point fixation using non-absorbable sutures placed in both the longitudinal and transverse axes. These cases underscore the fact that a high index of suspicion for RTT must be maintained regardless of prior surgical history or patient age. Finally, our series demonstrates that testicular torsion can occur at any age, even in middle-aged men decades after a primary orchidopexy. The ‘safe’ window of 12–25 years should not mislead clinicians into dismissing torsion as a differential diagnosis in older patients presenting with acute scrotal pain. Due to the rarity of testicular torsion after prior orchidopexy, the development of prospective studies is likely to be extremely difficult, if not impossible. As such, case reports and retrospective cohort studies play a crucial role in raising awareness among surgeons and patients about the potential for re-torsion and in guiding clinical decision-making. These forms of evidence are essential for informing conclusions regarding the most appropriate surgical techniques for orchidopexy. To reduce the risk of recurrence, future research efforts and surgical guidelines should explicitly address technical aspects of testicular fixation and promote standardized, evidence-based approaches. The main limitation of this paper is its descriptive nature and the small number of cases inherent to case reports.

## Data Availability

The original contributions presented in this study are included in the article. Further inquiries can be directed to the corresponding author.
